# 重组人血管内皮抑制素静脉持续泵入联合窗口期动脉灌注化疗治疗晚期肺鳞癌的临床观察

**DOI:** 10.3779/j.issn.1009-3419.2015.08.05

**Published:** 2015-08-20

**Authors:** 远 吕, 镕 姜, 春华 马, 金铎 李, 斌 王, 立伟 孙, 宁 穆

**Affiliations:** 300060 天津，天津市环湖医院肿瘤介入科，天津市脑血管与神经变性重点实验室 Department of Intervention, Tianjin HuanHu Hospital, Tianjin Key Laboratory of Cerebral Vascular and Neurodegenerative Disease, Tianjin 300060, China

**Keywords:** 肺肿瘤, 重组人血管内皮抑制素, 动脉灌注化疗, 不良反应, Lung neoplasms, Recombinant human vascular endostatin, Arterial infusion chemotherapy, Adverse reactions

## Abstract

**背景与目的:**

肺癌是我国最常见的恶性肿瘤之一，本研究旨在观察重组人血管内皮抑制素（恩度）静脉持续泵入联合窗口期动脉灌注化疗治疗晚期肺鳞癌患者的有效性及安全性。

**方法:**

2014年2月-2015年1月10例经细胞学或组织学病理确诊的Ⅲb期-Ⅳ期的肺鳞癌患者采用持续静脉泵入重组人血管内皮抑制素联合窗口期动脉灌注化疗与10例同期住院接受单纯动脉灌注化疗的Ⅲb期-Ⅳ期的肺鳞癌患者比较，联合治疗组重组人血管内皮抑制素剂量均为30 mg/d，持续静脉泵入，d1-d7，在第4天血管正常化窗口期接受动脉灌注化疗，灌注化疗采用多西他赛联合顺铂化疗方案，单纯治疗组动脉灌注化疗方案同联合治疗组。每4周为1周期，连续治疗2个周期后4周行近期疗效评价及药物不良反应评价。

**结果:**

两组患者均完成2次以上治疗，联合治疗组有效率（response rate, RR）为70.0%，疾病控制率（disease control rate, DCR）为90.0%，单纯治疗组RR为50.0%，DCR为70.0%，差异无统计学意义（*P*=0.650, *P*=0.582）。两组的药物不良反应轻微，主要为1级-2级胃肠道反应和血液毒性，差异无统计学意义（*P*=0.999, *P*=0.628）。联合治疗组1例患者发生1级心脏毒性。两组患者未发生3级以上药物不良反应。

**结论:**

重组人血管内皮抑制素静脉持续泵入联合窗口期动脉灌注化疗治疗晚期肺鳞癌近期疗效明显，患者耐受性良好，且不良反应轻微。

## Abstract (en)

**Background and objective:**

Lung cancer is one of the most common malignant tumors in China. The aim of this study is to observe the efficacy and safety of recombinant human vascular endostatin (endostar) durative transfusion combined with window period arterial infusion chemotherapy in the treatment of advanced lung squamous carcinoma.

**Methods:**

From February 2014 to January 2015, 10 cases of the cytological or histological pathology diagnosed stage Ⅲb -stage Ⅳ lung squamous carcinoma were treated with recombinant human vascular endostatin (30 mg/d) durative transfusion combined with window period arterial infusion chemotherapy. Over the same period of 10 cases stage Ⅲb -stage Ⅳ lung squamous carcinoma patients for pure arterial perfusion chemotherapy were compared. Recombinant human vascular endostatin was durative transfused every 24 hours for 7 days in combination group, and in the 4^th^ day of window period, the 10 patients were received artery infusion chemotherapy, using docetaxel combined with cisplatin. Pure treatment group received the same arterial perfusion chemotherapy regimen. 4 weeks was a cycle. 4 weeks after 2 cycles, to evaluate the short-term effects and the adverse drug reactions.

**Results:**

2 groups of patients were received 2 cycles treatments. The response rate (RR) was 70.0%, and the disease control rate (DCR) was 90.0% in the combination group; In the pure treatment group were 50.0%, 70.0% respectively, there were no statistically significant difference (*P*=0.650, 0.582). The adverse reactions of the treatment were mild, including level 1-2 of gastrointestinal reaction and blood toxicity, there were no statistically significant difference (*P*=0.999, *P*=0.628). In the combination group, 1 patient occurred level 1 of cardiac toxicity.

**Conclusion:**

Recombinant human vascular endostatin durative transfusion combined with window period arterial infusion chemotherapy in the treatment of advanced lung squamous carcinoma could take a significant curative effect, and the patients were well tolerated by the mild adverse reactions.

肺癌是我国最常见的恶性肿瘤之一，非小细胞肺癌（non-small cell lung cancer, NSCLC）约占所有肺癌的80%，肺鳞癌约占NSCLC的30%。以三代化疗药物联合铂类的两药化疗方案虽然提高了晚期肺鳞癌的疗效，但有效率仅为20%-30%，疗效进入了平台期^[1]^。随着分子靶向药物的出现，该类药物逐渐成为杀伤肿瘤的一个重要武器。重组人血管内皮抑制素注射液（恩度）是我国上市的多靶点血管内皮抑制剂，其通过抑制内皮细胞增殖及迁移，发挥抗血管生成的作用，从而促进肿瘤细胞凋亡。重组人血管内皮抑制素本身无细胞毒性作用，与化疗联合可达到疗效协同作用^[2]^。重组人血管内皮抑制素联合静脉化疗已经成为了NSCLC的一线治疗方案^[3, 4]^。

动脉灌注化疗是治疗晚期肺癌的一个非常有效的治疗方式，在提高疗效的同时减少了化疗药物带来的副反应^[5, 6]^。研究^[7]^报道，重组人血管内皮抑制素持续泵点联合窗口期（第4天）静脉化疗取得较好的临床效果。现通过观察天津市环湖医院肿瘤介入科自2014年2月-2015年1月应用重组人血管内皮抑制素持续静脉泵入联合窗口期（第4天）动脉灌注化疗治疗晚期肺鳞癌10例与单纯动脉灌注化疗治疗的晚期肺鳞癌10例比较，观察联合方案的有效性及安全性，现将结果报告如下。

## 1 资料与方法

### 1.1 病例资料

自2014年2月-2015年1月我科住院患者中共20例进入本研究，联合治疗组10例，男性8例，女性2例；中位年龄65岁（63岁-81岁）；ECOG功能状态评分为0分-1分3例，2分-3分7例；Ⅲb期5例，Ⅳ期5例，Ⅳ期患者以颅内、骨及肝转移多见。单纯治疗组10例，男性8例，女性2例；中位年龄68岁（60岁-83岁）；ECOG功能状态评分为0分-1分5例，2分-3分5例；Ⅲb期4例，Ⅳ期6例，Ⅳ期患者以颅内、骨及肝转移多见。两组患者的基线特征无统计学差异（均*P* > 0.05），具有可比性（表 1）。

**1:** 20例晚期鳞癌患者基线情况比较 The baseline comparison of 20 cases with adanced squamous carcinoma

Clinical features	Combined treatment group (cases)	Simple treatment group (cases)	*χ*^2^	*P*
Gender			0.001	0.999
Male	8	8		
Female	2	2		
Stage			0.202	0.999
Ⅲb	5	4		
Ⅳ	5	6		
PS score			0.833	0.650
0-1	3	5		
2-3	7	5		
PS: performance status.				

### 1.2 病例选择

纳入标准：①经组织病理学或细胞学确诊的Ⅲb期-Ⅳ期肺鳞癌患者；②肺部病灶经胸部计算机断层扫描（computed tomography, CT）诊断及评价，至少有1个可测量的病灶；③既往未接受过抗肿瘤治疗的，且拒绝接受外科手术、静脉化疗、放射治疗者；④东部肿瘤协作组（Eastern Cooperative Oncology Group, ECOG）功能状态评分为0分-3分；⑤预计生存期≥12周；⑥血常规及肝肾功能等化验指标和心功能基本正常，无重要器官出血、功能障碍；⑦能够耐受动脉灌注化疗；⑧签署知情同意书。排除标准：①有出血倾向、血栓病史或服用抗凝药物；②脏器功能异常，无法耐受重组人血管内皮抑制素及化疗副作用。

### 1.3 治疗方法

联合治疗组患者在治疗前均置入深静脉置管，使用百特输液泵静脉持续输注重组人血管内皮抑制素，剂量为30 mg/d，d1-d7。在第4天血管正常化窗口期接受动脉灌注化疗。本组患者灌注化疗均采用多西他赛60 mg-80 mg联合顺铂60 mg-80 mg动脉灌注化疗方案。动脉灌注化疗采用Seldinger穿刺技术，经一侧股动脉穿刺，用导管分别选择性插入支气管动脉及其他转移灶供血动脉内，经数字剪影血管造影（digital subtraction angiography, DSA）确定为肿瘤供血动脉，经导管依次灌注经0.9%生理盐水20 mL-40 mL稀释的化疗药物。单纯动脉灌注化疗组患者仅接受动脉灌注化疗治疗，动脉灌注化疗过程同联合治疗组。每4周为1个周期，连续治疗2个周期后行评价有效性及安全性。

### 1.4 近期疗效评价

根据实体瘤疗效评价标准（Response Evaluation Criteria in Solid Tumors, RECIST）1.1版标准对患者进行疗效评价，分为完全缓解（complete response, CR）、部分缓解（partial response, PR）、疾病稳定（stable disease, SD）和疾病进展（progressive disease, PD），有效率（response rate, RR）=（CR+PR）/总例数×100%，疾病控制率（disease control rate, DCR）=（CR+PR+SD）/总例数×100%。

### 1.5 药物不良反应的评价

所有患者在2个治疗周期前后进行血常规、肝肾功能、心肌酶、心电图及影像学检查以评价安全性。根据美国国立癌症研究所制订的通用药物毒性反应标准（National Cancer Institute-Common Toxicity Criteria, NCI-CTC）4.0版分级标准，对治疗期间或随访期间出现的各种不良事件进行评价。

### 1.6 统计学方法

采用SPSS 17.0软件进行统计与分析。以χ^2^检验比较两组患者基线水平、近期疗效、药物不良反应发生率，理论数小于5时采取*Fisher*确切概率法，*P* < 0.05为差异有统计学意义。

## 2 结果

### 2.1 近期疗效

两组患者均完成2个周期治疗后4周行近期疗效评价。联合治疗组10例患者中CR 0例，PR 7例，SD 2例，PD 1例，RR为70.0%，DCR为90.0%。单纯治疗组10例患者中CR 0例，PR 5例，SD 2例，PD 3例，RR为50.0%，DCR为70.0%。两组的RR和DCR差异无统计学意义（均*P* > 0.05）（表 2）。

**2:** 两组患者近期疗效的比较 The comparison of two groups in the immediate curative effect

Group	Cases	CR+PR	*χ*^2^	*P*	CR+PR+SD	*χ*^2^	*P*
Combined treatment group	10	7 (70%)	0.833	0.650	9 (90%)	1.250	0.582
Simple treatment group	10	5 (50%)			7 (70%)		
CR: complete response; PR: partial response; SD: stable disease.							

### 2.2 不良反应

两组20例患者均经股动脉穿刺成功率达100.0%，动脉灌注化疗术中未发生药物过敏等不良反应，术后未出现皮下血肿、假性动脉瘤等并发症。化疗药物相关的不良反应轻微，联合治疗组主要为1级-2级胃肠道反应30.0%（3/10）及血液毒性20.0%（2/10），本组未发生3级以上化疗药物不良反应。单纯治疗组主要为1级-2级胃肠道反应40.0%（4/10）及血液毒性40.0%（4/10）。两组胃肠道反应（χ^2^=0.220, *P*=0.999）及血液毒性（χ^2^=0.952, *P*=0.628）差异均无统计学意义。联合治疗组患者均未发生与深静脉置管相关的并发症，如深静脉血栓形成。重组人血管内皮抑制素主要的不良反应有心脏毒性、出血风险、高血压、蛋白尿等，本组仅有1例患者在接受第2周期治疗时出现1级心脏毒性，予对症治疗后好转，无其他严重不良反应。

## 3 讨论

重组人血管内皮抑制素是我国自主研发的一种新型血管内皮抑制剂，2005年9月被SFDA批准上市，2006年被《NCCN非小细胞肺癌临床实践指南（中国版）》收录，具有广谱抗肿瘤血管生成活性。重组人血管内皮抑制素是一种与细胞外基质胶原羧基末端具有同源性的内源性糖蛋白，它通过抑制内皮细胞一氧化氮合成酶的激活、阻断血管内皮生长因子（vascular endothelial growth factor, VEGF）介导的信号转导及抑制bcl-2/bcl-XL、bad表达促进内皮细胞凋亡等多种途径阻断血管生成，从而起到抑制肿瘤细胞生长的作用^[8]^。研究^[9]^表明重组人血管内皮抑制素使骨桥蛋白的表达下调, 进而诱导前基质金属蛋白酶（matrix metalloproteinase, MMP）-2、MMP-9的表达下降, 抑制肿瘤转移。研究^[10]^显示血管内皮抑素通过下调bcl-2蛋白表达，诱导肿瘤细胞凋亡。自2013年开始，美国国立综合癌症网络（National Comprehensive Cancer Network, NCCN）关于NSCLC的临床实践指南指出^[11]^：对于表皮生长因子受体（epidermal growth factor receptor, EGFR）及*ALK*突变未明或野生型的NSCLC患者，推荐使用重组人血管内皮抑制素联合化疗控制肿瘤进展。多项报道^[2, 12, 13]^显示：与单纯化疗比较，重组人血管内皮抑制素联合化疗能提高总有效率及临床收益率，并且降低11%的白细胞减少的风险，但在包括心脏毒性等其他不良反应方面没有差异。“血管正常化”一词是由Jain等^[14]^在2005年提出的：肿瘤内部的血管都是畸形异常的，从而导致血管渗透压的改变，组织内流体静力压升高，导致化疗药物不能正常进入肿瘤细胞内。血管内皮抑素可以改善修复肿瘤组织内部杂乱的血管网，使血管正常化，降低组织间压力，让化疗药物可以更直接的进入到组织内，杀伤肿瘤细胞。重组人血管内皮抑制素能让肿瘤血管系统暂时正常化，达到“血管正常化时间窗”^[15]^。此时化疗药物能更容易的进入到肿瘤组织中去，从而更好的达到杀灭肿瘤的治疗效果。研究^[7, 16]^表明重组人血管内皮抑制素的血管正常化时间窗出现在用药后的3 d-5 d，在此时间段内给予化疗药物，能获得最佳治疗效果。重组人血管内皮抑制素常规的给药方式是每天静点4 h，连续使用14 d，在一定程度上降低了患者的依从性和生活质量，最近有研究^[7, 12]^报道重组人血管内皮抑制素24 h持续静脉泵入的给药方式，可明显提高患者的依从性和生活质量，却不增加患者的不良反应，具有更好的耐受性。

以三代化疗药物联合铂类的两药方案化疗是治疗晚期肺鳞癌的标准方案，但疗效进入了平台期。动脉灌注化疗是治疗晚期肺癌的有效方式之一。动脉灌注化疗可经导管将化疗药物直接送达肿瘤供养动脉，药物总剂量约为全身静脉化疗剂量的1/3，从而大大提高了患者对化疗药物的耐受性，且可以同时治疗肺部原发灶及转移灶，明显提高患者的中位生存期^[5, 6]^。许健等^[17]^将40例NSCLC患者分成重组人血管内皮抑制素联合GP方案双途径给药治疗组（试验组）和重组人血管内皮抑制素联合单纯GP方案单途径治疗组（对照组），两组均行GP方案动脉灌注化疗，结果显示试验组无进展生存时间有所延长，但总体生存期改善不明显，两组均未出现严重不良反应。

本组研究基于上述理论，通过观察重组人血管内皮抑制素静脉持续泵入联合窗口期（第4天）动脉灌注化疗治疗晚期肺鳞癌10例患者与同期住院单纯行动脉灌注化疗治疗的10例患者比较，评价其近期疗效及安全性。结果显示联合治疗组患者的有效率和疾病控制率均高于单纯治疗组，但差异未达到统计学意义。联合治疗组患者的不良反应低于单纯治疗组，但差异无统计学意义。联合治疗组有1例患者在行第2周期重组人血管内皮抑制素泵入时出现了一过性的心悸，心电图显示为窦性心动过速，予对症药物处理后好转，未再出现心悸症状。我们认为重组人血管内皮抑制素可以提高动脉灌注化疗疗效，降低化疗毒副反应，且自身毒副反应轻微，患者的远期疗效值得期待。由于本研究样本量小，数据说服力相对较差，需要扩大病例数进一步研究探讨。
