# 更正声明

**DOI:** 10.3779/j.issn.1009-3419.2016.03.08

**Published:** 2016-03-20

本刊2015年第18卷第8期刊登的题为“重组人血管内皮抑制素静脉持续泵入联合窗口期动脉灌注化疗治疗 晚期肺鳞癌的临床观察”[吕远, 姜镕, 马春华, 李金铎, 王斌, 孙立伟, 穆宁. 重组人血管内皮抑制素静脉持续泵入 联合窗口期动脉灌注化疗治疗晚期肺鳞癌的临床观察. 中国肺癌杂志, 2015, 18(8): 500-504.]一文中，第503页“自 2013年开始，美国国立综合癌症网络（National Comprehensive Cancer Network, NCCN）关于NSCLC的临床实践指 南指出^[11]^：对于表皮生长因子受体（epidermal growth factor receptor, EGFR）及ALK突变未明或野生型的NSCLC患 者，推荐使用重组人血管内皮抑制素联合化疗控制肿瘤进展。 ”应删除。特此更正。对此深表歉意。 

Erratum: Clinical Observation of Recombinant Human Vascular Endostatin Durative Transfusion Combined with Window Period Arterial Infusion Chemotherapy in the Treatment of Advanced Lung Squamous Carcinoma

Yuan LV, Rong JIANG, Chunhua MA, Jinduo LI, Bin WANG, Liwei SUN, Ning MU

ZhongguoFei Ai ZaZhi, 2015, 18(8): 500-504.

In the version of this article initially published, error appeared on page 503. The sentence“自2013年开始，美国国立综合癌 症网络（National Comprehensive Cancer Network, NCCN）关于NSCLC的临床实践指南指出^[11]^：对于表皮生长因子受体（epidermal growth factor receptor, EGFR）及ALK突变未明或野生型的NSCLC患者，推荐使用重组人血管 内皮抑制素联合化疗控制肿瘤进展。 ”should bedeleted.

## Notes

https://www.ncbi.nlm.nih.gov/pmc/articles/PMC6000236/

